# Ultra-Trace Blood Concentrations of Blood Serum Arsenic and Breast, Prostate and Colorectal Cancer Risks in the EPIC-Heidelberg Cohort

**DOI:** 10.3390/cancers18030511

**Published:** 2026-02-04

**Authors:** Maureen Kipkoech, Jan Lubiński, Wojciech Marciniak, Róża Derkacz, Theron Johnson, Rudolf Kaaks, Verena A Katzke

**Affiliations:** 1 Division of Cancer Epidemiology, German Cancer Research Center (DKFZ), 69120 Heidelberg, Germany; maureen.kipkoech@dkfz.de (M.K.); t.johnson@dkfz.de (T.J.); r.kaaks@dkfz.de (R.K.); 2 Medical Faculty Heidelberg, Heidelberg University, 69120 Heidelberg, Germany; 3 Department of Genetics and Pathology, Pomeranian Medical University, 70-204 Szczecin, Poland; jan.lubinski@pum.edu.pl (J.L.); wojciech.marciniak@read-gene.com (W.M.); roza.derkacz@read-gene.com (R.D.); 4 Read-Gene SA, 72-003 Grzepnica, Poland

**Keywords:** serum arsenic, breast cancer, prostate cancer, colorectal cancer, EPIC Heidelberg

## Abstract

**Background/Objectives:** Background: The International Agency for Research on Cancer has classified arsenic as a group 1 carcinogen of the lung, skin and bladder. Arsenic has been implicated in the pathogenesis of breast, prostate, and colorectal cancers; however, existing evidence is limited and inconsistent. Prospective studies, particularly those employing blood-based quantification of arsenic, remain scarce. This study aimed to address this gap by investigating the association between serum arsenic levels and breast, prostate, and colorectal cancers in the European Prospective Investigation into Cancer and Nutrition (EPIC) Heidelberg case–cohort. **Methods**: Serum arsenic levels were measured using inductively coupled mass spectrometry in 5360 participants aged 35–65 years, recruited between 1994 and 1998. Over a median follow-up of 18 years (IQR: 17–19), 685 incident cases of breast, 597 of prostate, and 284 of colorectal cancer occurred. Prentice-weighted Cox proportional hazards regression models with age as the underlying time scale were used to estimate hazard ratios and confidence intervals for associations between serum arsenic levels and cancer risk. **Results**: No statistically significant association was found between serum arsenic levels and breast cancer, either overall with HR 1.04 (95% Cl: 0.96–1.35) or in subgroups based on pre- and post-menopausal status, estrogen/progesterone status, or BMI. Similarly, serum arsenic levels were not statistically associated with prostate cancer of HR 0.91 (95% Cl: 0.72–1.14). In contrast, a significant association with colon cancer emerged in the second quartile with HR 0.12 (95% Cl: 0.02–0.61) and third quartile with HR 0.19 (95% Cl: 0.05–0.73) compared to the first quartile but not in rectal cancer. **Conclusions**: More comparative studies on the different arsenic media and arsenic speciation should be conducted to determine the impact of arsenic on these cancers.

## Simple Summary

Arsenic has been hypothesized to cause breast, prostate and colorectal cancers, although there is a scarcity of evidence. The existing studies have mainly used food frequency questionnaires to measure arsenic exposure and associate with these cancers, even though that is a less reliable method. This study was designed to measure blood arsenic among participants in the European Prospective Investigation into Cancer and Nutrition (EPIC) Heidelberg case–cohort and investigate its association with the three cancers. Broadly, there was no association between serum arsenic levels and breast, prostate and colorectal cancers.

## 1. Introduction

Breast, prostate and colorectal cancers remain among the most pressing global health challenges, with substantial morbidity. The former is the most common cancer among women worldwide, with about 2.3 million new cases and over 685,000 deaths in 2020 [1]. Its development is influenced by a wide spectrum of risk factors spanning reproductive history, endogenous hormone metabolism, exogenous hormone use, environmental, lifestyle factors specifically BMI and smoking, and medical domains [2,3,4]; however, environmental factors have been less extensively studied [5,6,7]. Of particular concern are endocrine-disrupting chemicals (EDCs), which can interfere with hormone signaling pathways vital for breast tissue development and tumor growth [8]. Studies indicate that arsenic exposure may play a role in the development of breast cancer [9,10].

Prostate cancer is currently the second most frequently diagnosed cancer in men worldwide [1]. Despite advances in diagnosis and treatment, the etiology of prostate cancer remains only partially understood. A growing body of evidence suggests that disease development reflects a multifaceted interaction between age, genetics, family history, smoking, diet, physical activity, occupational factors, and certain medications like corticosteroids [11]. Increasing attention has focused on the role of potentially toxic elements (PTEs), which may disrupt prostate cell function leading to cancer [12]. Colorectal cancer represents the third most common global cancer diagnosis [1]. Established modifiable risk factors include smoking, unhealthy diet, low fruit and vegetable intake, high alcohol intake, overweight, and physical inactivity [13]. Emerging evidence implicates arsenic exposure as a contributor to prostate and colorectal cancer development [14,15].

Long-term exposure to inorganic arsenic is a significant public health concern in regions with frequent arsenic contamination of drinking water, including parts of South Asia, Latin America, and certain United States areas [16]. The International Agency for Research on Cancer [17] classified arsenic as a Group 1 carcinogen in 2012, with strong evidence linking it to skin, lung, bladder, and liver cancers among individuals in highly contaminated or occupational settings [17]. Despite these known connections, its link to breast, prostate and colorectal cancers have been less explored and even less so in lowly contaminated settings, leaving a significant gap in understanding low-dose exposure scenarios. Arsenic exposure in humans can be assessed through multiple pathways, including urine, blood, hair, nails and Food Frequency Questionnaire (FFQ). Blood arsenic levels are a promising biomarker for recent, integrated exposure to arsenic from both diet and environment. Unlike urine, which reflects short-term arsenic intake mainly influenced by seafood consumption (containing non-toxic organic arsenicals), blood measurements better represent the internal dose relevant for toxicity and are less confounded [18].

Few epidemiological studies have directly examined the association between blood arsenic and breast, prostate or colorectal cancer risk. To date, only one prospective epidemiological study has investigated the relationship between blood arsenic levels and breast cancer risk, conducted in Poland. It reported that baseline blood arsenic exposure was associated with a 13-fold increase in breast cancer risk among women with a family history of the disease [10]. However, whether high blood arsenic levels similarly increase the risk of primary breast cancer in women without family history of breast cancer remains unclear, underscoring the need for population-based research to clarify arsenic’s potential role in breast cancer development. Existing epidemiological studies investigating the associations of blood arsenic concentrations with risks of prostate and colorectal cancers are also limited, as they primarily focus on arsenic concentrations in drinking water and dietary intake which are unreliable indicators due to their inability to estimate internal dose levels. Although arsenic exposure can be measured through various methods, most previous studies have relied on FFQ to estimate dietary arsenic exposure and examine its association with these cancers. This approach is unreliable, highlighting the need for more reliable assessments. This study aims to investigate the relationship between blood arsenic levels and breast, prostate and colorectal cancers in the European Prospective Investigation into Cancer and Nutrition (EPIC) Heidelberg cohort.

## 2. Materials and Methods

### 2.1. Study Characteristics

This research used a case–cohort design within the EPIC-Heidelberg cohort. EPIC-Heidelberg serves as one of the study centers for the European Prospective Investigation into Cancer and Nutrition, a large-scale cohort study that enrolled over 520,000 participants across ten European countries. Participants have been followed for up to 20 years to examine the relationships between dietary, metabolic, and lifestyle factors and the risk of cancer as well as other chronic diseases [19]. The EPIC Heidelberg cohort comprises 25,546 participants (53% women; *n* = 13,617), aged 35–65 years, recruited between 1994 and 1998 from Heidelberg and surrounding regions. At baseline, information was collected on health, diet, lifestyle, socioeconomic status, and reproductive history via questionnaires and interviews using standardized and validated instruments [20,21,22]. Anthropometric measurements, including height, weight, waist, and hip circumference, were measured. Anthropometric examinations were undertaken by trained observers using standardized methods, and calipers, anthropometers and digital scales were controlled for measurement accuracy and technical correctness on a regular basis [23]. Blood samples were taken on the day of baseline recruitment, regardless of fasting, and stored at +4 °C to +10 °C for up to 24 h before processing. Samples were centrifuged and fractionated into plasma, serum, erythrocytes, and buffy coat, then stored in liquid nitrogen at −196 °C. Serum samples were used for the measurement of blood arsenic levels. Written informed consent was obtained from all participants at baseline [19].

The study endpoints were breast, prostate and colorectal cancers. Incident cases were identified through active follow-up of participants (directly or via next of kin) with systematic verification of self-reported cancer using clinical records, and via linkage to cancer and pathology registries. For this case–cohort analysis, we included all verified incident cases of breast (International Classification of Disease (ICD)-10: C50; *n* = 685), prostate (ICD-10: C61; *n* = 597), and colorectal cancer (ICD-10: C18–20; *n* = 284) diagnosed up to December 2014. For breast cancer, tumor estrogen receptor (ER) and progesterone receptor (PR) status was available. Participants with unavailable blood sample were excluded and those with previous other malignant tumors, except non-melanoma skin cancer, were censored at date of the previous tumor diagnosis. The comparison group is a representative subset of the full cohort, referred to as the sub-cohort.

The procedures for laboratory measurements are described in Appendix A.1 and in reference [19]. Measurement accuracy and precision were validated with a certified reference material (CRM), Clincheck Plasmonorm Serum Trace Elements Level 1. Cancer cases and sub-cohort non-cases were randomly measured, with laboratory personnel blinded to group allocation. The coefficient of variation (CV%) for intrabatch Quality Control (QC) 1 and interbatch QC2 was 4.23% and 5.05%, respectively, indicating good precision.

### 2.2. Sub-Cohort Sampling

**Figure A1:**
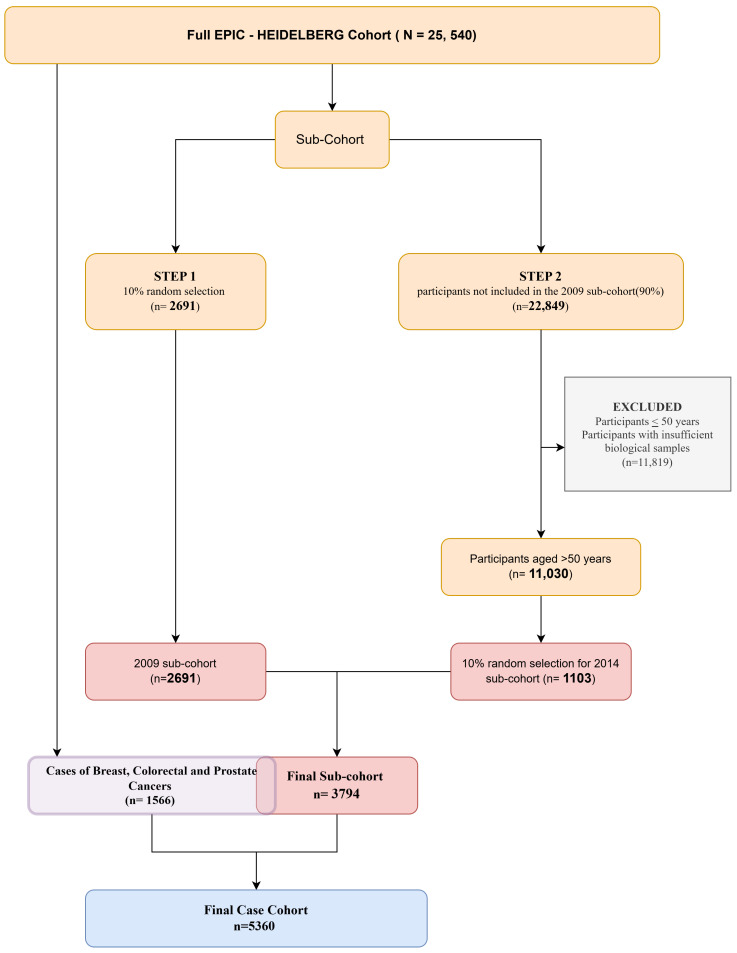
Graphical representation of the selection process of the EPIC-Heidelberg Case–Cohort.

As illustrated in Appendix A Figure A1, a two-stage, age-stratified case–cohort sampling strategy was applied within the EPIC-Heidelberg cohort to optimize efficiency in specimen use and enable robust investigation of age-related outcomes. Case–cohort designs minimize unnecessary use of biological specimens and financial costs for laboratory analysis while allowing the study of multiple outcomes [24]. To enhance statistical power for analyses of age-related effects, the design intentionally oversampled older participants in the sub-cohort, in accordance with higher cancer occurrences among older individuals. In the first sampling stage (2009), a 10% random selection of the entire baseline cohort was undertaken. These participants were included in the initial case–cohort analyses of chronic disease cases diagnosed up to December 2009 [25,26]. In the second stage (2014), an additional 10% sample was drawn from participants who were aged >50 years at baseline and had not been included in the first stage. The two samples were combined to form the final sub-cohort of 3794 randomly selected participants. In addition to the sub-cohort, 1566 incident cases of breast, prostate, and colorectal cancer diagnosed and verified through the end of December 2014 were included in this analysis. For the current analyses, 3794 sub-cohort study participants and 1566 incident cases were included.

### 2.3. Statistical Analysis

Baseline characteristics of cases and the non-case sub-cohort were summarized as median (interquartile range, IQR) for continuous covariates, and as frequencies and percentages for categorical covariates, and were further stratified by serum arsenic levels. We first assessed the association between serum arsenic concentrations and potential main factors of breast, prostate and colorectal cancers using a survey-weighted generalized linear model (GLM) with 95% confidence intervals (CI). For the main analyses of serum arsenic and the incidence of breast, prostate, and colorectal cancers, we used Prentice-weighted Cox proportional hazards regression models with age as the underlying time scale to estimate hazard ratios (HRs) and 95% Cls. Both crude and multivariable-adjusted HRs were calculated. All sub-cohort observations were left-truncated at age at recruitment and right-censored at the earliest of end of follow-up, death, or loss to follow-up. Cancer cases were included at the time of diagnosis, following the Prentice weighting scheme, with deaths from other causes accounted for.

Serum arsenic concentration was modeled in quartiles, with the lowest category as the reference group, because arsenic values did not follow a normal distribution. Covariates were retained in the multivariable models if they altered the HRs by more than 10%, were significantly associated with either the exposure or the outcome, or were considered important based on biological plausibility. The adjusted model for prostate and colorectal cancers included level of education (primary, secondary, technical/professional, tertiary), smoking status (never, former, current, unknown), alcohol use (never, former, current at recruitment, lifetime), body-mass index (BMI, continuous), and physical activity (inactive, moderately inactive, moderately active, active) as the covariates. In the breast cancer model, adjustments were made for additional covariates such as parity status (nulliparous, primiparous, multiparous), use of oral contraceptives or hormone replacement therapy (HRT), breastfeeding history (yes, no), age at menarche (<12 years, 12–14, >14 years), and family history of breast cancer (yes, no). Subgroup analyses were per formed to assess associations between serum arsenic and breast cancer stratified by menopausal status at baseline and at diagnosis, by tumor receptor status (ER/PR-positive vs. ER/PR-negative), and by BMI. Values below the limit of detection (LOD ≤ 0.022 µg/L) were retained in the main analysis to avoid inflating blood serum arsenic estimates. Sensitivity analysis was further performed using LOD/2 for values below the detection limit to confirm the robustness of our findings. An additional analysis was equally conducted to adjust for arsenic dietary proxies and verify the accuracy of the results. All statistical analyses were conducted in R (version 4.5.1, R Project for Statistical Computing, RRID:SCR_001905, Posit Software (R version 4.5.1), PBC, Boston, MA, USA). The software is available under the GNU Affero General Public License version 3 (AGPL v3).

## 3. Results

### 3.1. Participant Characteristics

A total of 144 samples were excluded as they were designated for quality control, and an additional 153 samples for being below the detection limit (≤0.022 µg/L) due to clogging, leaving a total of 4939 viable samples. The final study population included 3567 sub-cohort members, 645 breast cancer cases, 565 prostate cancer cases, and 272 colorectal cancer cases. One additional subject was excluded due to an implausibly high serum arsenic level of 258 µg/L, resulting in a final sub-cohort of 3566 participants. Table 1 summarizes the baseline characteristics of the EPIC-Heidelberg Case–Cohort participants. The median follow-up time in the sub-cohort was 18 years (IQR: 17–19), and the median age at recruitment was 54 years (IQR: 47–59). The sub-cohort comprised a slightly higher proportion of women (52%) compared to men (48%). The median age at recruitment was 55 years (IQR 49;59) for men and 53 years (IQR 44;58) for women. A comparable proportion of both men and women (63% each) had attained a technical or tertiary level of education at the time of recruitment. Among men, approximately half (42%) were former smokers, whereas a higher proportion of women (54%) reported never having smoked. Majority of the participants reported being lifetime alcohol drinkers.

### 3.2. Serum Arsenic Levels Stratified by the Baseline Characteristics

**Figure A2:**
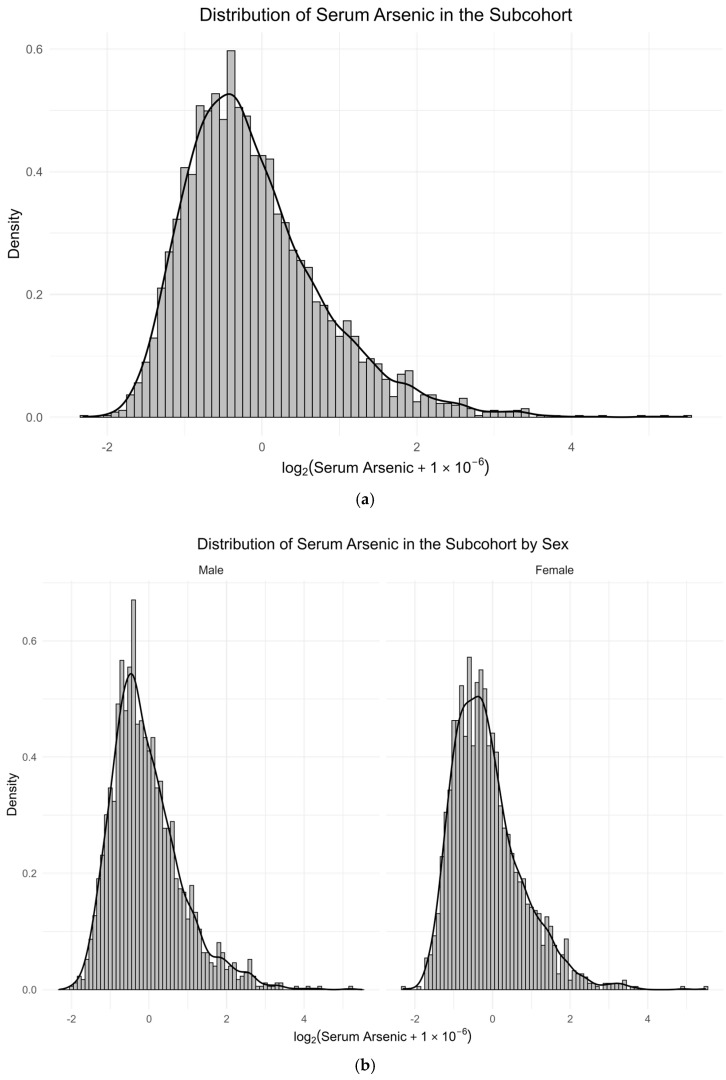
(**a**) Histogram with an overlaid density plot showing the distribution of blood serum arsenic levels among subcohort participants. (**b**) Histogram with an overlaid density plot showing the distribution of blood serum arsenic levels among subcohort participants, by sex.

The distribution of serum arsenic levels overall and by sex is shown in Appendix A Figure A2a,b. Serum arsenic levels were slightly higher among men, with the highest median concentration observed in the ≥60 age group (0.88 µg/L), compared to women across all age categories, as shown in Table 2. In terms of BMI status, serum arsenic distribution differed between sexes: among men, the highest levels (0.92 µg/L) were observed in the underweight group, while among women, the highest concentrations (0.90 µg/L) were found in the obese group. Regarding the level of education, the highest serum arsenic levels were observed among both men and women who had a tertiary level of education (0.87 µg/L). In terms of smoking status, former smokers reported the highest serum arsenic levels among both men and women (0.85 µg/L). With respect to alcohol usage, men that drank only at recruitment demonstrated the highest serum arsenic levels (0.85 µg/L), while among women, both drinkers at recruitment and lifetime alcohol drinkers exhibited the highest concentrations (0.83 µg/L). 

### 3.3. Factors Associated with Serum Arsenic Levels

As shown in Table 3, we used a general linear model to evaluate cancer-related factors associated with serum arsenic levels in the sub-cohort. A one-unit increase in BMI was significantly associated with a 0.26% decrease in serum arsenic. In contrast, age was positively associated with serum arsenic levels, with each year increase in age corresponding to a 0.19% increase in arsenic. Similarly, alcohol consumption was positively associated with serum arsenic, with each additional g/d linked to a 0.13% increase. Level of education demonstrated a graded effect: individuals in the tertiary level category had serum arsenic levels 4.7% higher than those in the primary category. Family history of breast cancer was also a significant predictor, with women reporting a second-degree relative affected exhibiting 8.6% higher serum arsenic levels compared to those with first-relative degree relatives. By contrast, parity status, breastfeeding, and oral contraceptive/HRT use were not significantly associated with serum arsenic levels in the adjusted model.

### 3.4. Associations Between Serum Arsenic Levels and Breast, Prostate and Colorectal Cancers

Table 4 presents the associations between serum arsenic levels and breast cancer risk. Using a crude Prentice-weighted Cox proportional hazards model, we observed that serum arsenic appeared protective in the second and third quartiles compared to the lowest quartile, with hazard ratios of HR_Q2 vs. Q1 = 0.86 (95% CI: 0.58–1.29) and HR_Q3 vs. Q1 = 0.86 (95% CI: 0.58–1.27), respectively. In contrast, participants in the highest quartile exhibited a slightly higher risk, HR_Q4 vs. Q1 = 1.1 (95% CI: 0.76–1.61). After adjusting for relevant covariates, the same pattern was observed, with the highest quartile showing a modestly elevated risk relative to the lowest quartile (HR: 1.04; 95% CI: 0.71–1.52). None of these associations reached statistical significance.

Exploratory analyses of menopausal status at baseline and at diagnosis (Appendix A Table A1 and Table A2) revealed variable patterns across quartiles among menopausal groups (premenopausal and postmenopausal). In general, the highest quartile was associated with an increased risk of breast cancer, although the HRs were not statistically significant. Specifically, the HRs were as follows: premenopausal at recruitment, HR 1.11 (95% CI: 0.86–1.45); postmenopausal status at recruitment, HR 1.02 (95% CI: 0.82–1.28); premenopausal status at diagnosis, HR 1.15 (95% CI: 0.76–1.73); and postmenopausal status, HR 1.03 (95% CI: 0.87–1.21).

Additional subgroup analyses by breast cancer receptor and BMI status exhibited patterns similar to those observed in the other subgroup analysis (Appendix A Table A3 and Table A4). With respect to prostate cancer as illustrated in Table 4, higher serum arsenic levels were non-significantly associated with an increased risk of prostate cancer. Specifically, comparing the highest to the lowest quartile, the HR was 1.13 (95% Cl: 0.95–1.34) in the basic model and 1.14 (95% Cl: 0.96–1.35) in the adjusted model; however, these associations did not reach statistical significance. Interestingly, levels of serum arsenic showed a non-significant inverse association with risk of colorectal cancer with both crude and adjusted HRs for the highest vs. lowest quartile being 0.91 (95% Cl: 0.72–1.14). As provided in Appendix A Table A5, subgroup analysis by colorectal site revealed a statistically significant protective association between blood arsenic levels and colon cancer in the second and third quantiles, with effect sizes of HR 0.12 (95% Cl: 0.02–0.61) and 0.19 (95% Cl: 0.05–0.73), respectively. Additional analysis using LOD/2 for values below the detection limit and adjusting for dietary arsenic proxies yielded results consistent with the main findings, as shown in Appendix A Table A6 and Table A7, respectively.

## 4. Discussion

In our population-based prospective study, serum arsenic levels were not statistically significantly associated with the risks of breast, prostate, or colorectal cancers after adjusting for potential confounders.

In the current study, we were unable to establish a statistically significant association between serum arsenic exposure and breast cancer, which is paradoxical to previous findings. A comparative Polish prospective cohort found that elevated whole blood arsenic levels increased breast cancer risk (HR: 13.2; 95% Cl 4.78–37) in women with a family history of the disease [10]. Arsenic exposure has been shown to aggravate DNA double-strand break (DSB) repair deficiencies, particularly in cells deficient in BRCA1 and BRCA2 [27,28]. This suggests that individuals harboring mutations in BRCA1 or BRCA2 may be more susceptible to arsenic-induced impairments in DNA repair. The Polish study proposed that a likely mechanistic explanation for their observed association is that arsenic mimics estrogen-induced effects, contributing to breast cancer [29]. If this mechanism applied broadly, similar results would have been expected in our study. We therefore hypothesized that arsenic exposure may be more hazardous in DNA-deficient or compromised individuals, which could explain why our population-based study did not identify a significant association, in contrast to the Polish study, which focused specifically on women with a family history of the disease. Consistently, the mean serum arsenic concentration in our cohort was 1.27 µg/L, substantially lower than the mean level of 1.62 µg/L reported in whole blood in the Polish cohort [10]. Similarly, findings from other perspective studies, which employed various exposure media such as airborne, FFQ, and drinking water also demonstrated a positive association between arsenic exposure and breast cancer [30,31,32,33]. However, there have been previous prospective studies using airborne or toenail-derived arsenic that reported null findings, which align with the results of the present study on blood levels [34,35]. The observed inconsistencies may be attributed to differences in population characteristics, sample size, exposure pathways, arsenic species, and laboratory techniques employed.

Our study did not find a statistically significant association between serum arsenic exposure and prostate cancer risk, in contrast to a meta-analysis that reported a significant association, with a pooled relative risk of 1.18 (1.06–1.30) [36]. Notably, none of the included studies employed blood samples; rather, the investigations relied on urine, drinking water, estimated intake from diet, and soil as the exposure materials. However, studies conducted in Denmark and North Carolina reported a non-significant association between arsenic exposure and prostate cancer risk, which is consistent with our findings [37,38]. Variations in arsenic assessment may account for the differing results, as most previous studies employed exogenous source of exposure, whereas the present study utilized endogenous arsenic.

Evidence regarding colorectal cancer remains limited and inconclusive. Overall, no statistically significant association was observed between blood arsenic levels and colorectal cancer in our study. However, subgroup analysis by colorectal site revealed that higher blood arsenic levels were statistically protective against colon cancer in the second and third quantiles relative to the first. Arsenic metabolites may remain longer in contact with the colonocytes, since colon has a longer absorption transit time compared to rectum which could explain these findings. A Danish prospective cohort found no clear association between drinking water arsenic exposure and colorectal cancer, consistent with our general results [37]. In contrast, evidence from Argentina demonstrated sex-specific effects, with elevated arsenic in drinking water associated with increased colorectal cancer risk in women but a protective effect in men [39]. The limited available evidence has primarily used drinking water as the exposure biomarker, which may explain the discrepancies in results. Moreover, the limited evidence suggesting a lack of association between arsenic exposure and colorectal cancer indicates that arsenic exposure is unlikely to be a major driver of the disease, particularly in areas with low-to-moderate exposure.

Arsenic, a naturally occurring metalloid, exists in organic and inorganic forms in the environment [40]. Human exposure to arsenic can occur through various sources, including drinking water, food, industrial processes, and tobacco smoke [41]. The more toxic inorganic arsenic is often found in contaminated groundwater, industrial emissions, and foods like rice and seafood [42]. The influence of these exposures on blood arsenic levels remains unclear, as no published guidelines have been established for permissible arsenic concentrations in the blood. Even so, evidence from clinical and epidemiological studies suggests a recommended threshold of 1 µg/L [43]. In vitro studies indicate that arsenic toxicity is influenced by the cells used in the treatment, the type of the animal model utilized, and the uptake rate of the arsenic compounds under investigation [44]. The total estimated arsenic intake from food and beverages generally ranges between 20 and 300 µg/day. However, it remains unclear whether elevated dietary intake directly correlates with increased arsenic levels in blood. Ambient inhalation of arsenic from air is typically a minor route of exposure for the general population, with estimated daily intakes of 20–200 ng for individuals in rural areas, 400–600 ng for residents of cities with arsenic-industrial emission activities, and 1 µg to over 10 µg for non-smokers and smokers, respectively, assuming a daily inhalation rate of 20 m^3^ [45,46].

Arsenic has been suggested to promote breast cancer carcinogenesis through mechanisms such as genotoxicity and DNA damage, chromosomal instability, and epigenetic changes [47,48,49]. Furthermore, laboratory studies suggest arsenic mimics estrogen and may act as an endocrine disruptor, particularly for hormone receptor-positive breast cancers and especially in tumor initiation and progression [50]. In prostate cancer, arsenic has been implicated in carcinogenesis through its ability to disrupt DNA repair mechanisms [15]. In colorectal cancer, in vitro studies have demonstrated that arsenic trivalent form and its metabolites may promote the disease through mechanisms involving oxidative stress, and proinflammatory response [51].

The absence of detectable association in this study may be attributable to serum arsenic levels in our cohort being insufficient (≤0.022 µg/L) to elicit measurable effects. Some samples of low volume were excluded, which could have influenced the overall sample composition. Specific concentrations of arsenic species, including inorganic arsenic, monomethyarsonic, dimethylarsinic or pentavalent arsenic, were not measured, as the inductively coupled mass spectrometer used did not allow for arsenic speciation despite the known differential effects of these species. Hence, results should be addressed with caution. Furthermore, the findings reflect short-term arsenic exposure, as arsenic has a short half-life and is cleared from blood within 3 to 6 h. Thus, serum arsenic levels rather reflect short- to mid-term rather than long-term exposure. An exception is continuous high-level exposure to inorganic arsenic from sources such as drinking water or food like fish, which can contribute to elevated arsenic blood levels, thereby reflecting the actual tissue burden. To better determine the influence of individual arsenic species on cancer risk, future studies should incorporate arsenic speciation. Repeated blood sample collection and measurement of arsenic levels around the time of diagnosis were not available but would allow further investigations into, so far, unknown areas. Despite these limitations, this study is the first large-scale prospective study to assess the effects of serum arsenic and risks of main cancers in a healthy population.

## 5. Conclusions

In conclusion, this prospective study found no evidence supporting an association between blood arsenic exposure and breast, prostate, or colorectal cancers. As the second blood-based arsenic prospective study, further research is warranted to clarify these hypothesized associations. Additionally, arsenic speciation analyses are needed to determine the potential effects of different arsenic metabolites on these cancers. A comparative analysis of arsenic levels across different biological media, for example, blood compared with urine, toenail and hair tissues, are warranted to evaluate potential associations between arsenic levels and site-specific cancers. Lastly, since this research was carried out in the EPIC-Heidelberg cohort, another implication for future research is the need for additional studies in diverse cohorts and populations worldwide to examine cancer outcomes.

## Figures and Tables

**Table 1:** Baseline Characteristics of the Sub-Cohort and of Incident Cancer Cases in the EPIC-Heidelberg cohort (media; IQR or N (%)).

Baseline Characteristic	Total Sub-Cohort	Men	Women	Breast Cancer	Prostate Cancer	Colorectal Cancer
	(*n* = 3566)	(*n* = 1730)	(*n* = 1836)	(*n* = 645)	(*n* = 565)	(*n* = 272)
Cancer cases in the sub-cohort	279	148	131	102	110	67
Age at recruitment	54 (47; 59)	55 (49; 59)	53 (44; 58)	54 (47; 58)	57 (54; 61)	58 (53; 61)
Age at diagnosis	-	-	-	61 (54; 66)	67 (64; 71)	65 (60; 69)
Length of follow-up	18 (17; 19)	18 (17; 19)	18 (17; 19)	17 (16; 19)	18 (17; 19)	18 (17; 19)
Body mass index	26 (23; 29)	27 (24; 29)	25 (22; 28)	24 (22; 28)	27 (25; 29)	28 (25; 29)
Level of Education						
Primary	1077 (30%)	542 (31%)	535 (29%)	163 (25%)	193 (34%)	100 (37%)
Secondary	220 (6%)	88 (6%)	132 (7%)	53 (8%)	15 (3%)	13 (5%)
Technical	1230 (34%)	469 (27%)	761 (41%)	257 (40%)	163 (29%)	88 (32%)
Tertiary	1039 (29%)	631 (36%)	408 (22%)	172 (27%)	194 (34%)	71 (26%)
Smoking Status						
Never	1574 (44%)	580 (34%)	994 (54%)	334 (52%)	234 (41%)	92 (34%)
Formerr	1214 (34%)	732 (42%)	482 (26%)	192 (30%)	232 (41%)	112 (41%)
Current	768 (22%)	413 (24%)	355 (19%)	117 (18%)	98 (17%)	67 (25%)
Alcohol Use						
Never drinker	57 (2%)	15 (1%)	42 (2%)	13 (2%)	3 (1%)	2 (1%)
Former drinker	120 (3%)	62 (4%)	58 (3%)	19 (3%)	21 (4%)	12 (4%)
Drinker at recruitment	40 (1%)	6 (0.4%)	34 (2%)	4 (1%)	1 (0%)	3 (1%)
Lifetime drinker	3348 (94%)	1646 (95%)	1702 (93%)	609 (94%)	540 (96%)	254 (93%)

**Table 2:** Median Arsenic Levels Stratified by Baseline Characteristics.

Characteristic	Total Sub-Cohort	Men	Women	Breast Cancer	Prostate Cancer	Colorectal Cancer
**Age at recruitment**						
<50	0.83 (0.59–1.23)	0.85 (0.62–1.29)	0.81 (0.56–1.20)	0.83 (0.60–1.19)	0.72 (0.57–1.05)	0.69 (0.59–0.97)
50–59	0.83 (0.60–1.30)	0.83 (0.62–1.30)	0.82 (0.58–1.30)	0.86 (0.59–1.35)	0.90 (0.65–1.52)	0.89 (0.61–1.18)
≥60	0.85 (0.61–1.34)	0.88 (0.61–1.39)	0.83 (0.62–1.30)	0.81 (0.65–1.26)	0.84 (0.60–1.53)	0.84 (0.61–1.33)
**BMI status**						
Underweight	0.86 (0.61–1.29)	0.92 (0.66–1.38)	0.84 (0.58–1.21)	0.86 (0.60–1.29)	0.83 (0.58–1.60)	0.88 (0.61–1.48)
Normal Weight	0.78 (0.57–1.21)	0.77 (0.57–1.19)	0.80 (0.57–1.22)	0.82 (0.62–1.43)	0.83 (0.60–1.25)	0.76 (0.55–1.09)
Overweight	0.83 (0.61–1.32)	0.84 (0.61–1.32)	0.81 (0.60–1.31)	0.80 (0.61–1.20)	0.86 (0.62–1.48)	0.84 (0.63–1.11)
Obese	0.86 (0.67–1.30)	0.68 (0.63–1.19)	0.90 (0.71–1.30)	0.64 (0.50–0.64)	–	1.24 (1.11–1.37)
**Level of education**						
Primary	0.80 (0.58–1.20)	0.84 (0.60–1.23)	0.78 (0.56–1.14)	0.76 (0.60–1.10)	0.79 (0.57–1.19)	0.76 (0.58–1.09)
Secondary	0.83 (0.59–1.29)	0.83 (0.62–1.28)	0.83 (0.58–1.29)	0.85 (0.60–1.35)	0.86 (0.68–1.37)	0.80 (0.63–1.11)
Technical	0.81 (0.61–1.27)	0.77 (0.61–1.39)	0.84 (0.60–1.21)	0.95 (0.66–1.28)	1.09 (0.66–1.88)	0.77 (0.63–1.18)
Tertiary	0.87 (0.63–1.38)	0.87 (0.63–1.39)	0.87 (0.63–1.35)	0.83 (0.60–1.31)	0.90 (0.60–1.79)	0.95 (0.62–1.51)
**Smoking status**						
Never	0.81 (0.59–1.27)	0.79 (0.61–1.28)	0.81 (0.58–1.26)	0.84 (0.64–1.19)	0.76 (0.56–1.34)	0.88 (0.60–1.30)
Former	0.85 (0.61–1.30)	0.85 (0.60–1.29)	0.85 (0.61–1.31)	0.79 (0.58–1.21)	0.84 (0.62–1.42)	0.84 (0.60–1.17)
Current	0.83 (0.60–1.27)	0.87 (0.64–1.35)	0.81 (0.58–1.20)	0.84 (0.61–1.35)	0.90 (0.64–1.65)	0.78 (0.61–1.10)
**Alcohol use**						
Never drinker	0.76 (0.58–1.02)	0.7 (0.43–1.17)	0.77 (0.59–0.96)	0.65 (0.58–0.93)	0.83 (0.83–0.83)	1.09 (0.86–1.13)
Former drinker	0.72 (0.53–1.15)	0.73 (0.51–1.16)	0.68 (0.57–1.03)	0.75 (0.67–0.99)	0.78 (0.54–1.44)	0.65 (0.60–0.94)
Drinker at recr.	0.84 (0.60–1.30)	0.85 (0.62–1.32)	0.83 (0.58–1.27)	0.83 (0.61–1.27)	0.86 (0.62–1.48)	0.84 (0.60–1.23)
Lifetime drinker	0.82 (0.50–1.01)	0.71 (0.51–0.90)	0.83 (0.51–1.01)	0.80 (0.52–1.16)	0.53 (0.47–0.68)	0.56 (0.46–0.67)

**Table 3:** Multivariate adjusted associations between cancer-related factors and serum arsenic levels in the EPIC-Heidelberg sub cohort (N = 3566).

Variable	Relative Change	Lower 95% CI	Upper 95% CI
Intercept	80.4912	62.1828	100.8663
BMI (continuous)	−0.2621	−0.5046	−0.0191
Age at recruitment	0.1911	0.0409	0.3415
Alcohol intake (g/d)	0.1312	0.0779	0.1846
Parity status (ref: Nulliparous)			
Primiparous	−2.5903	−7.6459	2.7421
Multiparous	−1.3697	−6.3421	3.8666
Grand multiparous	−0.9527	−14.4162	14.6287
Breastfeeding (ref: no)			
Yes	−0.0248	−3.7918	3.8896
Education (ref: Primary)			
Secondary	2.7817	−1.8465	7.6281
Technical/Professional	2.8401	0.0919	5.6637
Tertiary	4.7041	1.5888	7.9149
Oral contraceptive/HRT use (ref: no)			
Yes	2.8103	−0.3025	6.0203
Family history of breast cancer (ref: no)			
1st degree relative	−3.1490	−6.5259	0.3498
2nd degree relative	8.5983	1.3454	16.3702

The model was adjusted for body mass index, age at recruitment, alcohol intake, parity status, breastfeeding, education, use of oral contraceptives/HRT, and family history of breast cancer; were applicable.

**Table 4:** Association between Serum Arsenic Levels and Breast, Prostate, and Colorectal cancers in the EPIC-Heidelberg case–cohort (HR and 95%CI).

Cancer Type	Q1	Q2	Q3	Q4	*p*-Trend
**Breast cancer**					
Number of cases	145	172	166	162	–
Sub-cohort non-cases	434	429	434	437	–
Median arsenic (µg/L)	0.49	0.69	0.98	1.87	–
Crude model HR	Ref	0.86 (0.58–1.29)	0.86 (0.58–1.27)	1.10 (0.76–1.61)	0.38
Adjusted model HR	Ref	0.82 (0.54–1.23)	0.83 (0.56–1.23)	1.04 (0.71–1.52)	0.34
**Prostate cancer**					
Number of cases	141	140	119	165	–
Sub-cohort non-cases	406	405	405	405	–
Median arsenic (µg/L)	0.51	0.72	1.03	1.94	–
Crude model HR	Ref	1.04 (0.88–1.22)	1.55 (0.97–1.35)	1.13 (0.95–1.34)	0.29
Adjusted model HR	Ref	1.02 (0.87–1.20)	1.15 (0.98–1.36)	1.14 (0.96–1.35)	0.31
**Colorectal cancer**					
Number of cases	66	69	77	60	–
Sub-cohort non-cases	875	875	875	875	–
Median arsenic (µg/L)	0.50	0.71	1.00	1.91	–
Crude model HR	Ref	0.94 (0.73–1.20)	0.88 (0.69–1.11)	0.91 (0.72–1.14)	0.37
Adjusted model HR	Ref	0.95 (0.75–1.22)	0.87 (0.69–1.11)	0.91 (0.72–1.14)	0.45

The breast cancer model was adjusted for level of education, BMI, smoking status, alcohol use, parity status, use of oral contraceptives/HRT, breastfeeding history, age at menarche, and family history of breast cancer. For prostate and colorectal cancers, adjustments were made for level of education, BMI, alcohol use, smoking status and physical activity.

## Data Availability

The data that support the findings of this study are available from the corresponding author upon reasonable request.

## Abbreviations

The following abbreviations are used in this manuscript:

BMI	Body Mass Index
BRCA	Breast cancer susceptibility protein
CI	Confidence Interval
CRM	Certified Reference Material
CV	Coefficient of Variation
DSB	DNA double-strand break
EDC	Endocrine-Disrupting Chemicals
EPIC	European Prospective Investigation into Cancer and Nutrition
ER	Estrogen receptor
FFQ	Food Frequency Questionnaire
GLMs	Generalized Linear Models
HR	Hazard Ratio
HRT	Hormone Replacement Therapy
IARC	International Agency for Research on Cancer
ICD	International Classification of Disease
IQR	InterQuartile Range
QC	Quality control
PR	Progesterone Receptor
PTEs	Potentially Toxic Elements

## Appendix A

### Appendix A.1. A Detailed Description of Serum Arsenic Measurement

The collected serum was left at room temperature for at least 30 min but no longer than 2 h to facilitate clotting, then centrifuged at 1300 G for 12 min. Subsequently, the serum was transferred to cryovials and stored in a −80 °C freezer. On the day of testing, they were thawed, vortexed, and centrifuged at 5000 G for 5 min before measuring arsenic levels. Arsenic (75As) levels were measured using an inductively coupled mass spectrometer, Nexion 350D (PerkinElmer). Before each analysis, the instrument was tuned according to the manufacturer’s standards. Helium served as the collision gas. The spectrometer was calibrated with an external calibration approach. Standards were freshly prepared daily from a 10 µg/mL Multi-Element Calibration Standard 3 (PerkinElmer, Shelton, CT, USA), diluted with blank reagent to final concentrations of 0, 0.48, 0.99, and 1.98 µg/L for arsenic detection. Calibration curve correlation coefficients consistently exceeded 0.999. A matrix-matched calibration was used. The analysis involved a 40-fold dilution of serum with blank reagent, composed of high-purity water (>18 MΩ), TMAH (Alfa Aesar, Ward Hill, MA, USA), Triton X-100 (PerkinElmer), n-butanol (Merck, Rahway, NJ, USA), and EDTA (Sigma-Aldrich, Saint Louis, MO, USA).

**Table A1:** Association between Serum Arsenic Levels and Breast Cancer Risk by Menopausal Status at Baseline.

	Quartile of Arsenic				*p*-Trend
	Q1	Q2	Q3	Q4	
**Premenopausal**					
Number of cases	44	61	56	53	
Sub-cohort non-cases	151	145	150	150	
Median arsenic (µg/L)	0.47	0.67	0.93	1.80	
Crude HR (95% CI)	Ref	1.06 (0.80–1.41)	0.83 (0.63–1.09)	1.12 (0.86–1.45)	0.38
Adjusted HR (95% CI)	Ref	1.01 (0.76–1.35)	0.85 (0.64–1.11)	1.11 (0.86–1.45)	0.34
**Postmenopausal**					
Number of cases	81	79	76	82	
Sub-cohort non-cases	218	216	220	217	
Median arsenic (µg/L)	0.49	0.71	1.00	1.90	
Crude HR (95% CI)	Ref	0.98 (0.79–1.22)	1.06 (0.85–1.32)	1.05 (0.84–1.31)	0.96
Adjusted HR (95% CI)	Ref	0.93 (0.75–1.17)	1.10 (0.88–1.37)	1.02 (0.82–1.28)	0.78

The model was adjusted for level of education, BMI, smoking status, alcohol use, parity status, use of oral contraceptives/HRT, breastfeeding history, age at menarche, and family history of breast cancer.

**Table A2:** Association between Serum Arsenic Levels and Breast Cancer Risk by Menopausal Status at Diagnosis.

	Quartile of Arsenic				*p*-Trend
	Q1	Q2	Q3	Q4	
**Premenopausal**					
Number of cases	25	26	21	19	
Sub-cohort non-cases	456	455	455	456	
Median arsenic (µg/L)	0.49	0.69	0.98	1.87	
Crude HR (95% CI)	Ref	0.89 (0.59–1.36)	0.91 (0.60–1.38)	1.17 (0.78–1.76)	0.55
Adjusted HR (95% CI)	Ref	0.83 (0.54–1.27)	0.94 (0.62–1.43)	1.15 (0.76–1.73)	0.37
**Postmenopausal**					
Number of cases	120	146	145	143	
Sub-cohort non-cases	437	437	437	437	
Median arsenic (µg/L)	0.48	0.69	0.98	1.87	
Crude HR (95% CI)	Ref	1.05 (0.89–1.25)	0.94 (0.79–1.11)	1.04 (0.88–1.22)	0.82
Adjusted HR (95% CI)	Ref	1.01 (0.85–1.20)	0.95 (0.80–1.12)	1.03 (0.87–1.21)	0.88

The model was adjusted for level of education, BMI, smoking status, alcohol use, parity status, use of oral contraceptives/HRT, breastfeeding history, age at menarche, and family history of breast cancer.

**Table A3:** Association between Serum Arsenic Levels and Breast Cancer Risk by Receptor Type.

	Quartile of Arsenic				*p*-Trend
	Q1	Q2	Q3	Q4	
**ER/PR+**					
Number of cases	114	118	121	106	
Sub-cohort non-cases	442	442	442	442	
Median arsenic (µg/L)	0.48	0.69	0.98	1.87	
Crude HR (95% CI)	Ref	0.93 (0.77–1.12)	0.95 (0.79–1.14)	0.98 (0.82–1.18)	0.32
Adjusted HR (95% CI)	Ref	1.03 (0.87–1.23)	0.94 (0.79–1.11)	1.03 (0.87–1.21)	0.98
**ER/PR−**					
Number of cases	16	30	27	31	
Sub-cohort non-cases	454	454	453	454	
Median arsenic (µg/L)	0.49	0.69	0.99	1.87	
Crude HR (95% CI)	Ref	1.51 (0.99–2.29)	0.78 (0.52–1.16)	1.27 (0.88–1.85)	0.14
Adjusted HR (95% CI)	Ref	1.47 (0.96–2.25)	0.78 (0.52–1.16)	1.27 (0.87–1.86)	0.17

The model was adjusted for level of education, BMI, smoking status, alcohol use, parity status, use of oral contraceptives/HRT, breastfeeding history, age at menarche, and family history of breast cancer.

**Table A4:** Association between Serum Arsenic Levels and Breast Cancer Risk by BMI Status.

	Quartile of Arsenic				*p*-Trend
	Q1	Q2	Q3	Q4	
**≤24.9**					
Number of cases	93	87	86	87	
Sub-cohort non-cases	357	356	356	356	
Median arsenic (µg/L)	0.50	0.73	1.03	1.95	
Crude HR (95% CI)	Ref	0.94 (0.77–1.16)	1.04 (0.84–1.28)	1 (0.81–1.24)	0.14
Adjusted HR (95% CI)	Ref	0.98 (0.8–1.21)	0.99 (0.81–1.23)	1.03 (0.83–1.28)	0.99
**>25**					
Number of cases	63	93	66	70	
Sub-cohort non-cases	466	571	474	529	
Median arsenic (µg/L)	0.48	0.69	0.98	1.85	
Crude HR (95% CI)	Ref	0.93 (0.74–1.19)	0.91 (0.72–1.15)	1.12 (0.89–1.4)	0.48
Adjusted HR (95% CI)	Ref	0.95 (0.74–1.2)	0.89 (0.7–1.12)	1.16 (0.92–1.46)	0.45

The model was adjusted for level of education, smoking status, alcohol use, parity status, use of oral contraceptives/HRT, breastfeeding history, age at menarche, and family history of breast cancer.

**Table A5:** Association between Serum Arsenic Levels and Colorectal Cancer Risk by Colon Site.

	Quartile of Arsenic				*p*-Trend
	Q1	Q2	Q3	Q4	
**Colon cancer**					
Number of cases	43	41	54	29	
Sub-cohort non-cases	857	898	886	885	
Median arsenic (µg/L)	0.49	0.70	1.00	1.91	
Crude HR (95% CI)	Ref	**0.11 (0.02–0.59)**	**0.19 (0.05–0.71)**	0.44 (0.19–1.01)	0.06
Adjusted HR (95% CI)	Ref	**0.12 (0.02–0.61)**	**0.19 (0.05–0.73)**	0.44 (0.19–1.01)	0.07
**Rectal cancer**					
Number of cases	20	23	17	29	
Sub-cohort non-cases	865	903	892	881	
Median arsenic (µg/L)	0.49	0.70	1.00	1.91	
Crude HR (95% CI)	Ref	6.46 (0.62–66.77)	4.62 (0.59–35.96)	2.11(0.61–7.3)	0.21
Adjusted HR (95% CI)	Ref	5.58 (0.54–58.04)	3.94 (0.5–30.84)	1.94 (0.56–6.73)	0.20

The model was adjusted for level of education, BMI, alcohol use, smoking status and physical activity.

**Table A6:** Association between Serum Arsenic Levels (LOD/2) and Breast, Prostate, and Colorectal cancers.

Cancer Type	Q1	Q2	Q3	Q4	*p*-Trend
Breast cancer					
Number of cases	145	172	166	162	–
Sub-cohort non-cases	434	429	434	437	–
Median arsenic (µg/L)	0.49	0.69	0.98	1.87	–
Crude model HR	Ref	1.02 (0.87–1.19)	0.93 (0.80–1.08)	1.06 (0.91–1.24)	0.97
Adjusted model HR	Ref	0.96 (0.81–1.14)	0.97 (0.82–1.15)	1.05 (0.89–1.24)	0.61
Prostate cancer					
Number of cases	141	140	119	165	–
Sub-cohort non-cases	404	412	403	401	–
Median arsenic (µg/L)	0.50	0.72	1.03	1.94	–
Crude model HR	Ref	1.04 (0.88–1.22)	1.17 (0.99–1.38)	1.14 (0.96–1.36)	0.98
Adjusted model HR	Ref	1.02 (0.87–1.20)	1.17 (0.99–1.39)	1.15 (0.97–1.36)	0.59
Colorectal cancer					
Number of cases	66	69	76	61	–
Sub-cohort non-cases	873	878	872	875	–
Median arsenic (µg/L)	0.50	0.71	1.01	1.91	–
Crude model HR	Ref	0.95 (0.74–1.21)	0.89 (0.7–1.13)	0.91 (0.73–1.15)	0.98
Adjusted model HR	Ref	0.96 (0.75–1.23)	0.89 (0.7–1.13)	0.91 (0.72–1.15)	0.73

The breast cancer model was adjusted for level of education, BMI, smoking status, alcohol use, parity status, use of oral contraceptives/HRT, breastfeeding history, age at menarche, and family history of breast cancer. For prostate and colorectal cancers, adjustments were made for level of education, BMI, alcohol use, smoking status and physical activity.

**Table A7:** Sensitivity analysis of blood serum arsenic—breast, prostate and colorectal cancers associations adjusted for arsenic dietary proxies.

Cancer Type	Q1	Q2	Q3	Q4	*p*-Trend
**Breast cancer**					
Number of cases	145	172	166	162	–
Sub-cohort non-cases	434	429	434	437	–
Median arsenic (µg/L)	0.49	0.69	0.98	1.87	–
Crude model HR	Ref	1.02 (0.87–1.19)	0.93 (0.8–1.08)	1.06 (0.91–1.24)	0.97
Adjusted model HR	Ref	1.05 (0.89–1.24)	1.18 (1–1.39)	1.15 (0.97–1.37)	0.25
**Prostate cancer**					
Number of cases	141	142	117	165	–
Sub-cohort non-cases	404	412	403	401	–
Median arsenic (µg/L)	0.50	0.72	1.03	1.94	–
Crude model HR	Ref	1.04 (0.88–1.22)	1.17 (0.99–1.38)	1.14 (0.96–1.36)	0.39
Adjusted model HR	Ref	1.05 (0.89–1.24)	1.18 (1–1.39)	1.15 (0.97–1.36)	0.25
**Colorectal cancer**					
Number of cases	66	69	76	61	–
Sub-cohort non-cases	873	878	872	875	–
Median arsenic (µg/L)	0.50	0.71	1.01	1.91	–
Crude model HR	Ref	0.94 (0.74–1.21)	0.89 (0.7–1.13)	0.91 (0.72–1.15)	0.43
Adjusted model HR	Ref	1 (0.78–1.29)	0.89 (0.7–1.14)	0.91 (0.72–1.15)	0.72

Models were additionally adjusted for arsenic dietary proxies: rice, fish and shellfish, non-alcoholic beverages, and water, in addition to the afore-adjusted covariates.
